# Angelica sinensis extract inhibits RANKL-mediated osteoclastogenesis by down-regulated the expression of NFATc1 in mouse bone marrow cells

**DOI:** 10.1186/1472-6882-14-481

**Published:** 2014-12-12

**Authors:** Lingbo Kong, Qinpeng Zhao, Xiaodong Wang, Jinyu Zhu, Dingjun Hao, Chongfei Yang

**Affiliations:** Hong-Hui Hospital, Xi’an Jiaotong University College of Medicine, 710054 Xi’an, China; Institute of Orthopedic Surgery, Xijing Hospital, Fourth Military Medical University, 710054 Xi’an, China

**Keywords:** Angelica sinensis, Osteoclastogenesis, NFATc1, BMMs

## Abstract

**Background:**

Destructive erosion of bone or osteolysis is a major complication of inflammatory conditions such as rheumatoid arthritis (RA), periodontal disease, and periprosthetic osteolysis. Natural plant-derived products have received recent attention as potential therapeutic and preventative drugs in human disease.

**Methods:**

The effect of Angelica sinensis (AS) extract on RANKL-induced osteoclast differentiation was examined in this study. The osteoclast precursor cell line bone marrow macrophages (BMMs) was cultured and stimulated with RANKL followed by treatment with AS at several doses. Gene expression profiles of c-Fos, c-Jun, NFATc1, TRAP, and OSCAR were sequentially evaluated.

**Results:**

AS extract inhibited RANKL-mediated osteoclast differentiation in BMMs in a dose-dependent manner without any evidence of cytotoxicity. AS extract strongly inhibited p38, ERK, JNK, p65 phosphorylation and I-κB degradation in RANKL-stimulated BMMs. AS extract also inhibited the mRNA expression of c-Fos, c-Jun, NFATc1, TRAP, and OSCAR in RANKL-treated BMMs. Moreover, RANKL-induced c-Fos, c-Jun and NFATc1 protein expression was suppressed by AS extract.

**Conclusions:**

These results collectively suggested that AS extract demonstrated inhibitory effects on RANKL-mediated osteoclast differentiation in bone marrow macrophages *in vitro*, indicating that AS may therefore serve as a useful drug in the prevention of bone loss.

## Background

Osteoclasts are derived from hematopoietic stem cells, which are unique multinucleated cells responsible for bone resorption [1]. Osteoblasts and stromal cells express receptor activators of the nuclear factor-kB (NF-kB) ligand (RANKL) and macrophage colony-stimulating factor (M-CSF) [2]. M-CSF induces expression of RANKL receptor (RANK) as well as supports survival and proliferation in early stage precursors of osteoclast lineages in mouse bone marrow cells [3]. RANKL is a member of the tumor necrosis factor (TNF) family and binds to the RANK receptor expressed in osteoclast precursor cells [4]. The binding of RANKL and RANK on osteoclast progenitor cells triggers the activation of tumor necrosis factor receptor-associated factor 6 (TRAF6) [5] and subsequently the activation of NF-kB and mitogen-activated protein kinases (MAPKs), such as extracellular signal-regulated kinase 1/2 (ERK1/2), p38 and stressactivated protein kinase/c-Jun N-terminal kinase (SAPK/JNK) [6, 7]. Although more detailed mechanism is still expected to be unveiled, the major signaling events triggered upon RANK ligation include recruitment of TRAF6, the activation of transcription factors NF-κB, c-Fos, AP-1, and nuclear factor of activated T cells (NFATc1) [2, 5, 8–11]. NFATc1 is a downstream transcription factor in the RANKL/RANK signal pathway and as a key molecule of osteoclastogenesis, NFATc1 induces a series of osteoclast-specific genes, including tartrate-resistant acid phosphatase (TRAP), osteoclast-associated receptor (OSCAR) and cathepsin K −[12, 13]. c-Fos is also an essential transcription factor for osteoclastogenesis and positively regulates osteoclastogenesis via NFATc1 activation [14].

Many plant-derived natural products have been used in traditional medicine for the treatment of various diseases. Several compounds derived from natural products have been recently reported to possess inhibitory effects on osteoclast differentiation and function, leading to decreased bone loss in vivo [1]. Angelica sinensis (AS) has been used to regulate menstruation, an inflammatory syndrome, in Asia for thousands of years. Recently, a component of AS extract, ligustilide has been reported to regulate several extracellular signaling pathways, including ERK1/2, p38 and SAPK/JNK [15]. Additionally, our previous study demonstrated that AS extract could inhibit wear debris particles-induced bone resorption by attenuating proinflammatory cytokines [16]. However, the effects of AS extract on RANKL and M-CSF functions vital to osteoclast differentiation are not clarified. In this study, we aimed to investigate the effects of AS extract on signaling pathways involved in osteoclast differentiation, activation, and survival *in vitro.*

## Methods

### Herb preparation

Dry root slices of a popular Chinese herb, Angelica sinensis (Dang Gui), was obtained from Tong Ren Tang (Tong Ren Tang Group Co., Ltd.; Beijing, China) and extracted in water (85°C) for 4 h. The water-soluble fraction was cleared sequentially by centrifugation (3300 × g, 20 min, 4°C) and filtration through a 0.2 mm filter [17]. From10 g dry Angelica sinensis root, about 0.8 g yellow, powdery substance was recovered after lyophilization.

### Reagents and antibodies

Human RANKL and M-CSF was obtained from Peprotech (London, UK). The XTT assay kit was obtained from Roche (Indianapolis, IN, USA). Antibodies for c-Fos, c-Jun and nuclear factor of activated T cells 1 (NFATc1) were purchased from Santa Cruz Biotechnology (Santa Cruz, CA, USA), and Western blot antibodies for phosphor-p65, p-65, phosphor-ERK, ERK, phosphor-JNK, JNK, phosphor-p38, p38, and I-κB were from Santa Cruz Biotechnology Inc. (Santa Cruz, CA, USA); β-actin antibody was purchased from Sigma-Aldrich, Inc. (St. Louis, MO, USA).

### Osteoclast differentiation

All the animal work and approach have been approved by the IACUC of the Hong-Hui Hospital, Xi’an Jiaotong University College of Medicine and conducted strictly followed by “the institutional guidelines for the care and use of laboratory animals at the Jiaotong University College of Medicine”. Bone marrow cells were obtained by flushing the femurs and tibiae of 5-week-old ICR mice with α-minimum essential medium (α-MEM; Gibco BRL, Gaithersburg, MD, USA) and suspended in α-MEM supplemented with 10% fetal bovine serum (FBS; Gibco BRL, Gaithersburg, MD, USA). Non-adherent cells were collected and cultured for 3 days in the presence of M-CSF (20 ng/ml). Floating cells were discarded and adherent cells on dish bottoms were classified as bone marrow derived macrophages (BMMs). BMMs were seeded at 3.5 × 10^4^ cells/well in α-MEM/10% FBS, and were cultured in the presence of M-CSF (20 ng/ml) and RANKL (40 ng/ml) for 4 days in the presence or absence of AS extract. Osteoclasts were identified by staining for tartrate-resistant acid phosphatase (TRAP) activity, as described below. TRAP-positive multinucleated cells with greater than three nuclei were counted as osteoclasts. Cytotoxicity assay for AS extract treated BMMs were plated in 96-well plates at a density of 1 × 10^4^ cells/well in triplicate. Cells were treated with M-CSF (20 ng/ml) and increasing concentrations of AS extract were added to the mix. Cells were incubated for 3 days. After 3 days, XTT reagent (50 μl) was added to each well. Wells were incubated for 4 h. The optical density at 450 nm was analyzed with an ELISA reader.

### Clonogenic assay

RAW 264.7 cells were seeded in 48-well plates at a density of 3 × 10^3^cells/well in triplicate and cultured for 4 days in the presence of increasing concentrations of AS extract. After 4 days, the cells were fixed and stained with Hematoxylin Sigma-Aldrich, Inc. (St. Louis, MO, USA). Colonies with 50 or greater cells were counted.

### Real time RT-PCR analysis for c-Fos, c-Jun, NFATc1, TRAP, osteoclast-associated receptor (OSCAR)

Total RNA was isolated with TRIzol reagent (Invitrogen Inc., USA) per the manufacturer’s instructions. RNA (1 μg) was reverse transcribed using oligo dT primers (10 μg) and dNTPs (10 mM). The mixture was incubated at 65°C for 5 min, and cDNA was produced by incubating at 42°C for 50 min with first strand buffer (50 mM Tris–HCl, pH 8.3, 75 mM KCl, 3 mM MgCl_2_), 100 mM DTT, RNase inhibitor, and Superscript II reverse transcriptase (Invitrogen). The cDNA was amplified using the following primer sets: c-Fos, 5′-CTGGTGCAGCCCACTCTGGTC- 3′ (forward) and 5′-CTTTCAGCAGATTGGCAATCTC-3′ (reverse); c-Jun, 5′-ACT CGG ACC TTC TCA CGT CG- 3′ (forward) and 5′-TAG ACC GGA GGC TCA CTG TG −3′ (reverse); NFATc1, 5′-CTCGAAAGACAGCACTGGAGCAT-3′ (forward) and 5′-CGGCTGCCTTCCGTCTCATAG-3′ (reverse); TRAP, 5′-CTGGAGTGCACGATGCCAGCGACA-3′ (forward) and 5′-TCCGTGCTCGGCGATGGACCAGA-3′ (reverse); OSCAR, 5′-CTGCTGGTAACGGATCAGCTCCCCAGA-3′ (forward) and 5′-CCAAGGAGCCAGAACCTTCGAAACT-3′ (reverse); and GAPDH, 5′-ACCACAGTCCATGCCATCAC-3′ (forward) and 5′-TCCACCACCCTGTTGCTGTA- 3′(reverse). PCR was performed using the QuantiTect SYBR Green PCR kit (Qiagen) in triplicates according to the manufacturer’s instructions. Relative levels of c-Fos, c-Jun, NFATc1, TRAP, and OSCAR were normalized to GAPDH.

### Western blot analysis

BMMs or osteoclasts were lysed in a buffer containing 50 mM Tris–HCl, 150 mM NaCl, 5 mM EDTA, 1% Triton X-100, 1 mM sodium fluoride, 1 mM sodium vanadate, 1% deoxycholate, and protease inhibitors. The lysates were centrifuged at 14,000 × g for 20 min and supernatants were collected. Protein concentrations of supernatants were determined. Cellular proteins (30 μg) were resolved by 8–10% sodium dodecyl sulfate-polyacrylamide gel electrophoresis (SDS-PAGE) and were transferred to polyvinylidene difluoride membranes (Milipore, Bedford, MA, USA). Non-specific interactions were blocked with 5% skim milk for 2 h and were then probed with the appropriate primary antibodies. Membranes were incubated with the appropriate secondary antibodies attached to horseradish peroxidase, and immunoreactivity was detected with enhanced chemiluminescence reagents. Densitometric values were quantified for each band with the Image Pro-plus program version 4.0.

### Statistical analysis

All data are expressed as means ± standard deviation (SD). Statistical analysis was done using SPSS software package ver. 11.0 (SPSS, Chicago, IL); one-way ANOVA was used for comparison among the different groups. *Post hoc* testing of differences between groups was performed by using Duncan’s test when the ANOVA was significant. All results were considered to be significant at the 5% critical level (*P < 0.05*).

## Results

### Inhibition of osteoclast differentiation by AS extract

**Figure 1:**
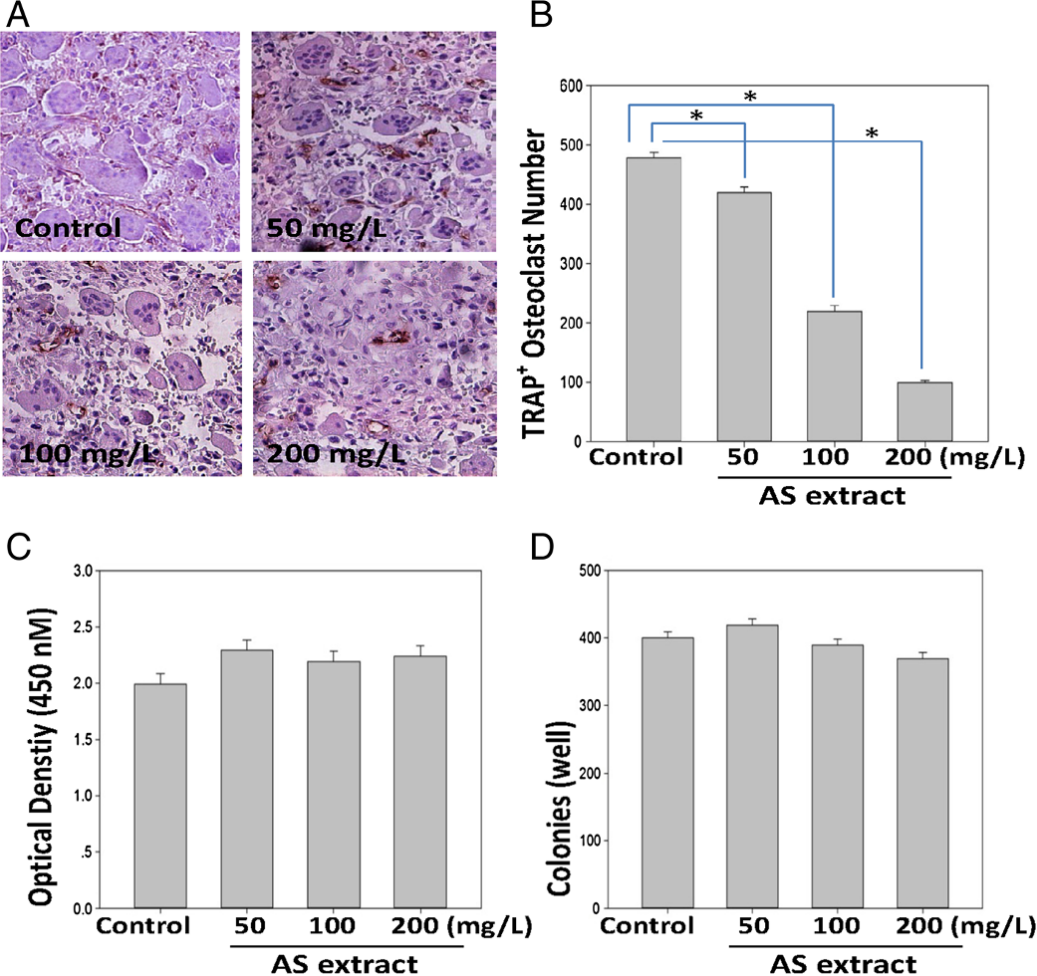
**Effect of AS extract on RANKL-induced osteoclast differentiation. (A)** BMMs were cultured for 4 days with M-CSF (20 ng/ml) and RANKL (40 ng/ml) in the presence of varying concentrations of AS extract. Cells were fixed with 3.7% formalin, permeabilized with 0.1% Triton X-100, and stained with TRAP solution. **(B)** TRAP-positive cells were counted as osteoclasts. Asterisk indicates a statistically significant difference (*p < 0.05*) between control and treated. **(C)** Cytotoxicity of AS extract on BMMs. BMMs were cultured for 3 days with varying concentrations of AS extract in the presence of M-CSF (20 ng/ml). XTT solution (50 μl) was added to each well after 3 days, and plates were incubated for 4 h. The optical density was measured at 450 nm. **(D)** RAW 264.7 cells were seeded at 3000 cells/plate in 48-well plates and cultured with the indicated concentrations of AS extract for 4 days. After 4 days, the cells were fixed and stained with Hematoxylin. Colonies with 50 or greater cells were counted. Similar results were obtained in at least 3 independent experiments.

Osteoclasts were generated from mouse BMMs in the presence of M-CSF (20 ng/ml) plus RANKL (40 ng/ml) to verify the effects of AS extract in osteoclastogenesis. The BMMs of the control group differentiated into mature TRAP-positive multinucleated osteoclasts while AS extract reduced the formation and numbers of TRAP-positive multinucleated cells in a dose-dependent manner (Figure 1A, B).

### The cytotoxic effect of AS extract

AS extract generated a highly negative effect on osteoclastogenesis. We measured the effects of AS extract on bone marrow cells with the XTT assay to exclude the possibility that the inhibition was due to cytotoxicity. AS extract demonstrated no cytotoxic effects at the same doses which effectively inhibited osteoclast formation (Figure 1C). Also, AS extract did not affect in RAW 264.7 cells colony formation (Figure 1D), suggesting that osteoclastogenesis suppression by AS extract was not due to toxic effects on BMMs.

### AS extract inhibits a variety of signals transduced by RANKL

**Figure 2:**
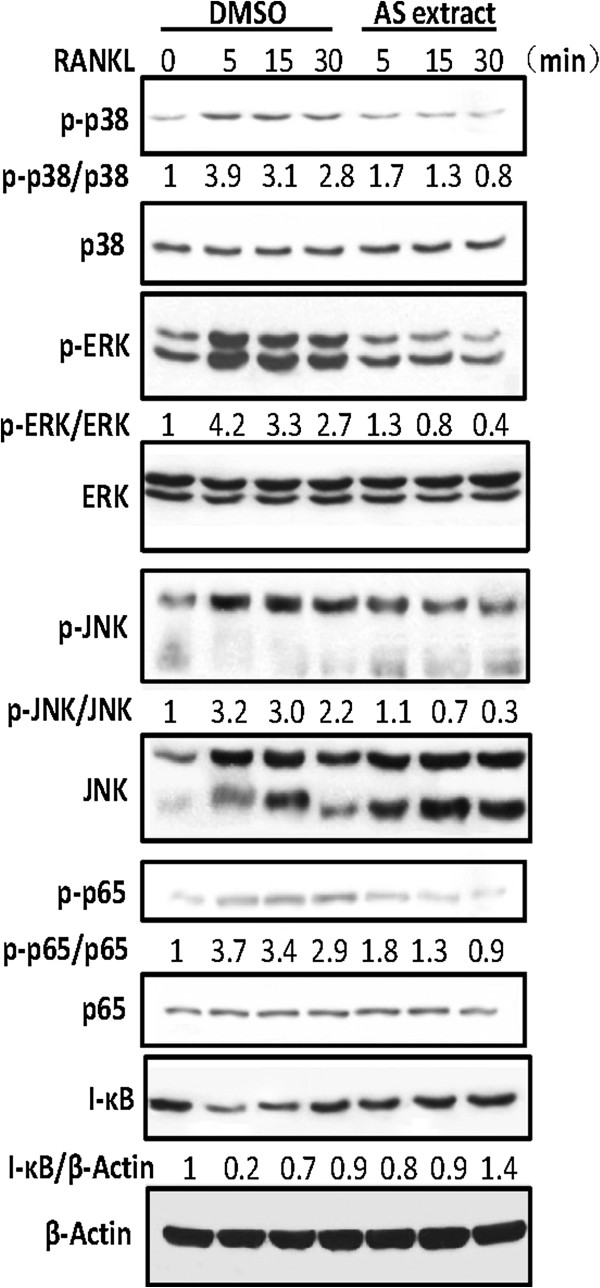
**Effect of AS extract on RANKL-induced MAPKs and NF-κB activation.** AS extract inhibits the p38, ERK, JNK, p65 phosphorylation and I-κB degradation in RANKL-stimulated BMMs. BMMs were pretreated with or without AS extract (50 mg/l) for 1 h prior to RANKL stimulation (100 ng/ml) at indicated time periods. Cells were lysed in lysis buffer, and lysates were analyzed by Western blotting with indicated antibodies. The intensities of protein bands were analyzed and normalized to actin. Similar results were obtained in at least 3 ndependent iexperiments.

RANKL activates a variety of signal transducers that are involved in osteoclastogenesis, including p38, JNK, ERK, transcription factor NF-κB and I-κB, which are recognized as the key factors of osteoclast differentiation [2, 3]. Osteoclast precursors were pretreated with AS extract and stimulated with RANKL at various time points. Different signaling pathways were observed. Activation of ERK, JNK, p38, NF-κB p65 and degradation of I-κB by RANKL were all significantly inhibited by AS extract (Figure2).

### RANKL induced c-Fos, c-Jun, NFATc1, TRAP, and OSCAR mRNA expression is reduced by AS extract

**Figure 3:**
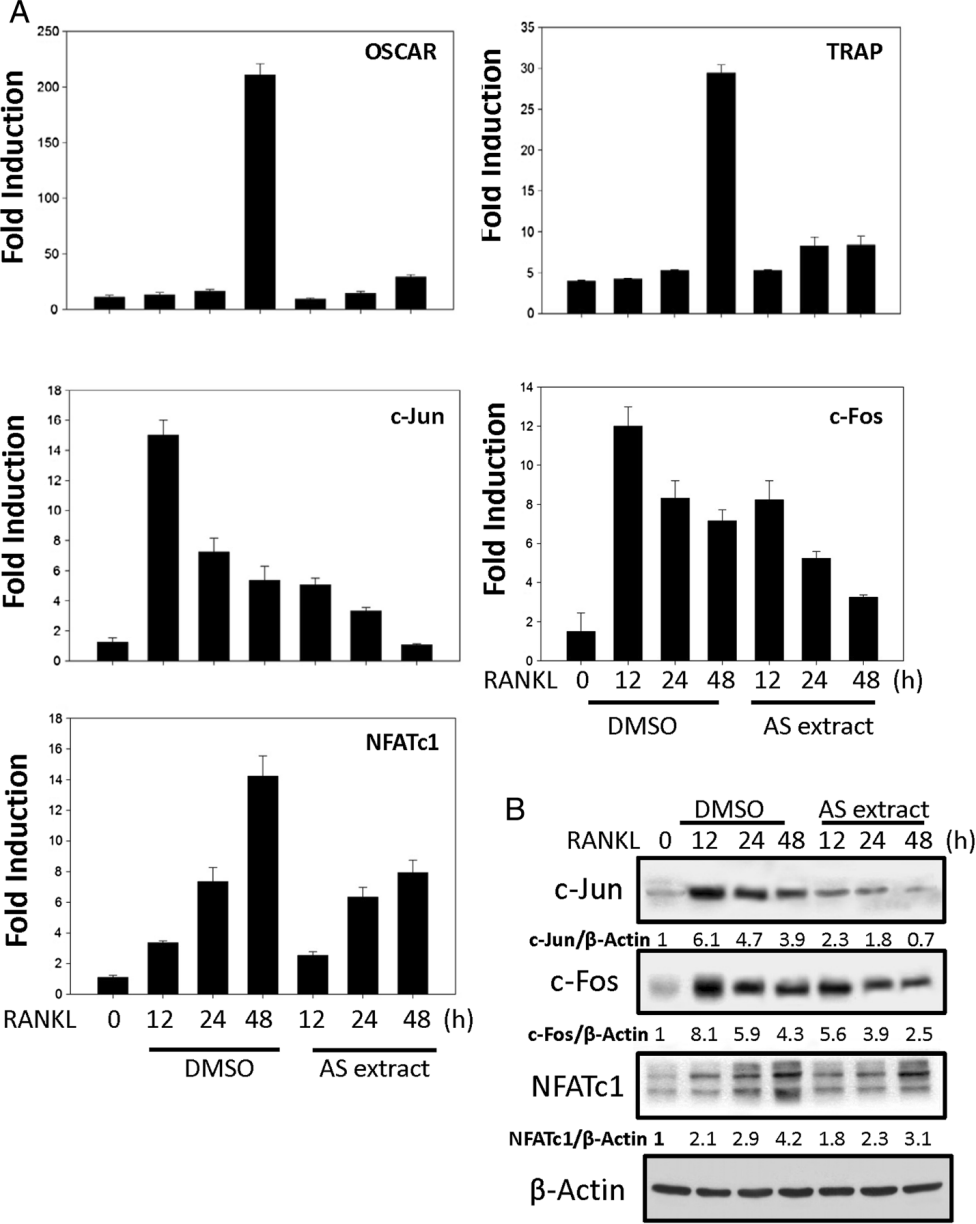
**AS extract suppresses the mRNA expression of c-Fos, c-Jun, NFATc1, OSCAR and TRAP in BMMs treated with RANKL. (A)** BMMs were pretreated with or without AS extract (50 mg/l) for 1 h and with RANKL (100 ng/ml) for the indicated time. Total RNA was isolated using TRIzol, and 1 μg of total RNA was used to transcribe cDNA. cDNA (1 μl) was used as a template for real time RT-PCR. The mRNA expression of the indicated genes was analyzed by real time RT-PCR. **(B)** AS extract inhibits the expression of c-Jun, c-Fos and NFATc1 induced by RANKL. BMMs were pretreated with or without AS extract (50 mg/l) for 1 h and were treated with RANKL (100 ng/ml) for the indicated time. Cells were lysed in the lysis buffer, and lysates were analyzed by Western blotting with antibodies against c-Jun, c-Fos, NFATc1 and actin. The intensities of the protein bands were analyzed and normalized to actin. Similar results were obtained in at least 3 independent experiments.

Osteoclast differentiation is regulated by the induction of various genes in response to RANKL and RANK binding. The c-Fos, c-Jun and NFATc1 genes have an essential role in the osteoclast differentiation [2, 13], and NFATc1 regulates OSCAR expression [18]. We examined the effects of AS extract on the RANKL-induced regulation of c-Fos, c-Jun and NFATc1 expression, and to assess whether there were any effects on TRAP and OSCAR expression. Osteoclast precursors were pretreated with AS extract and further stimulated with RANKL at various time points. Results revealed that c-Fos, c-Jun and NFATc1 mRNA levels were increased in response to RANKL, but c-Fos, c-Jun and NFATc1 mRNA expression was significantly inhibited by AS extract. Moreover TRAP and OSCAR mRNA expression was also significantly inhibited by AS extract (Figure 3A). This raises the possibility that, AS extract may inhibit osteoclast differentiation through the inhibition of RANKL-induced c-Fos, c-Jun and NFATc1 expression.

### AS extract also inhibits c-Fos, c-Jun and NFATc1 protein expression

Western blots were examined to verify the effects of AS extract on c-Fos, c-Jun and NFATc1 protein expression. c-Fos, c-Jun and NFATc1 protein levels were increased in response to RANKL, but c-Fos, c-Jun and NFATc1 expression was significantly inhibited by AS extract (Figure 3B). These results demonstrated that the inhibitory effects of AS extract involved the inhibition of major transcription factors such as c-Fos, c-Jun and NFATc1.

## Discussion

Bone resorption by osteoclasts is frequently caused by excessive RANKL signaling which has been a valuable target for the treatment of pathological bone loss. RANKL activates a series of major intracellular signal transducing pathways including NFATc1, NF-κB, phosphoinositide 3 kinase (PI3K)-Akt, JNK, ERK, and p38 MAPK. AS is one of the most popular Chinese herbs in both clinical field and research field. Su et al. [15] reported that a component of AS extract: ligustilide suppresses the LPS-induced production of NO, PGE2 and TNF-α by inhibiting both IKK/NF-κB and MAPK/AP-1 signaling. Recently we [16] demonstrated that AS extract could inhibit bone resorption by attenuating proinflammatory cytokines. However, the effect of AS extract on RANKL-induced osteoclast formation, especially RANKL-mediated intracellular signal pathway still is an interesting question that remains to be investigated.

The MAPK group of enzymes selectively phosphorylates serine and threonine residues in response to extracellular stimuli and transmits the stimuli from the cell surface to the nucleus [7]. MAPK is primarily composed of JNK, ERK, and p38 in mammalian cells. RANKL and RANK receptor binding expressed in osteoclast precursors provides a link between distinct signaling molecules such as JNK, ERK, and p38 MAPK [1]. p38 MAPK signaling is particularly crucial in the early stages of osteoclast differentiation as it promotes the activity of microphthalmia-associated transcription factor (MITF) and TRAP expression. Inhibition of p38 MAPK with SB203580 has a negative effect on osteoclast formation [19]. AS extract suppressed RANKL-induced the phosphorylation of p38, ERK and JNK activity in the present study (Figure 2).

The activation of signaling molecules induces transcription factors such as NF-κB, NFATc1, and AP-1 that are essential for osteoclast differentiation [20]. NF-κB is an important signal mediator for inflammatory and immune reactions and is a major transcription factor for RANKL-activated osteoclastogenesis [21]. I-κB is attached to NF-κB preventing it from migrating into the nucleus, and phosphorylation with I-κB kinase (IKK) separates the two proteins. Subsequent ubiquitination and proteosome degradation of I-κB allows the transfer of NF-κB into the nucleus and transcription of the target gene [22]. Although our previous study showed that one factor of AS extract: ligustilide decreases NF-κB luciferase activity in a reporter assay [16], we also confirmed that AS extract inhibits NF-κB/I-κB protein expression in this experiments. These results are consistent with RANKL activation of NF-κB in osteoclastic precursor cells through IKK activation and susequent I-κB phosphorylation and degradation (Figure2).

NFATc1 is a master regulator of osteoclastogenesis which autoamplifies and conducts the expression of osteoclast specific genes such as activator protein-1 (AP-1), TRAP, calcitonin receptor, OSCAR, and cathepsin K [10, 23–25]. The RANKL-induced NFATc1 expression is mediated by the activation of AP-1 consisting of c-Fos and c-Jun [26, 27]. c-Fos was reported to be critical for transcriptional activation of NFATc1 in RANKL-induced osteoclastogenesis [7]. Putative c-Fos binding sites were mapped in the promoter region of NFATc1, and the NFATc1 expression was abolished in the osteoclast precursors lacking c-Fos [11].On the other hand, Ikeda et al. [26] reported that RANKL could not induce expression of NFATc1 in dominant-negative c-Jun transgenic mouse. It is possible that the induction of NFATc1 may be cooperatively up-regulated by c-Fos and c-Jun, which make up each other for the induction [28]. The present study demonstrates the reduced both c-Fos and c-Jun expression in AS extract treated BMM cells (Figure 3). Therefore, the reduced c-Fos and c-Jun expression is suggested to cause the impaired activation of NFATc1, followed by the inhibition of RANKL-induced osteoclast formation. Additionally mRNA levels of major osteoclast marker TRAP and OSCAR was also inhibited by AS extract (Figure 3). These data suggest that the AP-1 transcription factor is the targets of AS extract-induced inhibition of osteoclastogenesis.

## Conclusions

In summary, the present study demonstrated that AS inhibited osteoclastogenesis from macrophages and bone resorption *in vitro*. AS also reduced the RANKL-induced expression of osteoclastic marker genes. In addition, AS attenuated RANKL-induced ERK, p38, JNK, NF-κB, AP-1 and NFATc1 activation. Although additional experiments are needed to confirm the efficacy of AS in treating disease conditions *in vivo*, our results indicate that AS has potential as a therapy for disorders associated with bone loss.
